# Biodegradable Polyurethanes Based on Castor Oil and Poly (3-hydroxybutyrate)

**DOI:** 10.3390/polym13091387

**Published:** 2021-04-24

**Authors:** Pathikrit Saha, Chanin Khomlaem, Hajer Aloui, Beom Soo Kim

**Affiliations:** Department of Chemical Engineering, Chungbuk National University, Cheongju, Chungbuk 28644, Korea; pathikritsaha89@gmail.com (P.S.); nament99@gmail.com (C.K.); hajer.aloui@ymail.com (H.A.)

**Keywords:** poly (3-hydroxybutyrate), castor oil, polyurethane, biodegradation

## Abstract

Biodegradable polyurethanes (PUs) were produced from castor oil (CO) and poly (3-hydroxybutyrate) diol (PHBD) using hexamethylene diisocyanate as a crosslinking agent. PHBDs of different molecular weights were synthesized through transesterification of bacterial PHB and ethylene glycol by changing the reaction time. The synthesized PHBDs were characterized in terms of Fourier transform infrared and proton nuclear magnetic resonance spectroscopy. A series of PUs at different NCO/OH and CO/PHBD ratios were prepared. The resulting CO/PHBD-based PUs were then characterized in terms of mechanical and thermal properties. Increasing PHBD content significantly increased the tensile strength of CO/PHBD-based PUs by 300% compared to neat CO-based PU. CO/PHBD-based PUs synthetized from short chain PHBD exhibited higher tensile strength compared to those produced from long chain PHBD. As revealed by scanning electron microscopy analysis, such improvement in stiffness of the resulting PUs is due to the good compatibility between CO and PHBD. Increasing PHBD content also increased the crystallinity of the resulting PUs. In addition, higher degradation rates were obtained for CO/PHBD-based PUs synthetized from long chain PHBD compared to neat CO PU and PUs produced from short chain PHBD.

## 1. Introduction

Polyurethanes (PUs) formed by the exothermic reaction between petroleum-based polyols containing two or more hydroxyl groups and isocyanates containing two or more isocyanate groups are the leading polymer families with an overall production ranked sixth around the world [1,2,3,4]. However, the extensive use of such petroleum-based polyols may lead to environmental pollution, and petroleum reserves depletion with a substantial increase in oil prices [5,6]. Therefore, scientists have been interested in the use of biobased resources for PU formulation. Recently, vegetable oils have been investigated as inexpensive alternatives to petroleum-based polyols [7,8].

Vegetable oils are mainly composed of triglyceride chains with a fatty acid triester of glycerol containing 12–22 carbon atoms and 0–3 carbon-carbon double bonds [5]. Among a variety of vegetable oils, castor oil (CO) has shown great potential in the green synthesis of PUs due to the presence of hydroxyl groups in its triglyceride chain [9,10]. Despite their good thermal stability, CO-based PUs exhibit poor mechanical properties and low biodegradation rate which may limit their application in many fields [10,11].

Poly(3-hydroxybutyrate) (PHB), an aliphatic polyester produced via bacterial fermentation, has drawn a considerable attention due to its biocompatibility and biodegradability [9]. However, due to its inherent brittleness, the use of PHB may be restricted in wide applications. To overcome this limitation, different soft segments including polyethylene glycol (PEG), polycaprolactone (PCL), polybutylene adipate and polyglycerol sebacate, were incorporated into PHB-based PUs [12,13,14,15,16]. Several additives such as PCL, soy filler, isosorbide, glycerol, cellulose, PEG, and molasses have been used with the aim to improve the mechanical properties of CO-based PUs along with increasing their biodegradation rate [11,16,17,18]. Chen et al. [19] prepared a CO-based polyurethane urea from CO and 4-aminophenyl disulfide and reported an increase in the mechanical properties and reprocessibility of the resulting films. A short chain fluorine containing chain extender 2,2,3,3-tetrafluoro-1,4-butanediol was used for the preparation of CO-based PU and a decrease in the tensile properties of the resulting films was observed as a result of increasing chain extender content [20]. In another work, cellulose acetate modified with methylene diphenyl diisocyanate (MDI) was used as a hard segment for the improvement of mechanical and thermal properties of CO-based PU [21]. Overall, the incorporation of such bio-additives may increase the hydroxyl numbers which may enhance the crosslinking of the polymers and thereby increase urethane groups. Urethane, amides, and esters are reported to be vulnerable to microbial attack and can be hydrolyzed to smaller molecules [11,18].

In this study, various end-capped PHB diols (PHBDs) of different molecular weights were synthesized through transesterification of bacterial PHB and ethylene glycol by changing the reaction time. Using PHBDs with CO as a polyol, new types of biodegradable biobased PUs were prepared using hexamethylene diisocyanate (HMDI) as a crosslinking agent. A series of CO/PHBD-based PUs were produced at different NCO/OH and CO/PHBD ratios. The resulting CO/PHBD-based PUs were then characterized in terms of mechanical and thermal properties. The effect of the molecular weight of the synthesized PHBDs on the properties of PU was also assessed. In addition, a soil burial biodegradation assay was carried out to evaluate the biodegradation rate of the prepared PUs.

## 2. Materials and Methods

### 2.1. Materials

Castor oil, chloroform, p-toluenesulfonic acid (p-TSA), sodium hydrogen phosphate (Na_2_HPO_4_ × 12H_2_O), ammonium sulfate ((NH_4_)_2_SO_4_), magnesium sulfate (MgSO_4_), dipotassium phosphate (KH_2_PO_4_), calcium chloride (CaCl_2_), sodium chloride (NaCl), glucose, and ethylene glycol (99%) were purchased from Samchun Chemicals (Korea). 1,6-hexamethylene diisocyanate and dibutyltin dilaurate were supplied from Sigma-Aldrich. All reagents used in this study were of analytical grade.

### 2.2. PHB Production and Extraction

The PHB polymer used for the synthesis of end-capped PHB diols in this study was produced by *Cupriavidus necator* KCTC 2649 as previously described by Khomlaem et al. [22]. Briefly, a minimal medium containing 1 g/L of (NH4)_2_SO_4_, 9 g/L of Na_2_HPO_4_ × 12H_2_O, 1 g/L of MgSO_4_ × 7H_2_O, 0.5 g/L of KH_2_PO_4_, 20 mg/L of CaCl_2_ × 2H_2_O, 10 g/L of NaCl, and 2 mL/L of trace element solution supplemented with 20 g/L of glucose was used for *C. necator* KCTC 2649 cultivation in shake flasks. The produced PHB polymer was extracted from lyophilized cells using hot chloroform as a solvent according to the method described by Fiorese et al. [23] with some modifications. Briefly, 1 g of freeze-dried bacterial cells was suspended in 100 mL of hot chloroform (60 °C) for 2 h under continuous stirring. The resulting suspension was filtered through Whatman No.1 filter paper. The filtrate was then poured into glass Petri plates and kept for 24 h under a chemical laminar flow hood for complete solvent evaporation. The recovered polymer was characterized in terms of Fourier transform infrared (FTIR) and proton nuclear magnetic resonance (1H-NMR) analysis.

### 2.3. Preparation of PHB Diol

PHBDs of different molecular weights were synthesized by transesterification of the purified bacterial PHB and ethylene glycol using p-TSA as a catalyst [13]. Briefly, 1 g of the purified PHB was dissolved in chloroform and refluxed under nitrogen atmosphere. After complete dissolution, 2 g of ethylene glycol and 0.48 g of p-TSA were added in the solution and the reaction temperature was set at 60 °C. The transesterification reaction was carried out at 2 h intervals for 8 h. After the reaction, the resulting solutions were rinsed 4 times with deionized water and the organic phase was separated and dried at room temperature to obtain PHBDs of different molecular weights. The resulting PHBDs were designated as PHBD 2h, PHBD 4h, PHBD 6h, and PHBD 8h.

### 2.4. Preparation of CO/PHBD-Based Polyurethane (CO/PHBD PU)

CO was mixed with the synthesized PHBD 8h in chloroform at different CO/PHBD weight ratios of 9/1, 8/2, 7/3, and 6/4, respectively. After thorough mixing, HMDI and dibutyltin dilaurate (0.1% *v/w*) used as crosslinking agent and catalyst, respectively, were added to the mixture. Afterwards, the mixture was continuously stirred for 1 h at room temperature. An excess of chloroform was added to dilute the obtained viscous reaction mixture. The resulting solution was poured in polytetrafluoroethylene plate and degassed under vacuum for 5–10 min at 50–60 °C before being cured at 80 °C for 24 h. Different NCO/OH molar ratios from 1.0 to 4.0 were used throughout the reaction. The effects of both NCO/OH and CO/PHBD ratios on the functional properties of the resulting CO/PHBD PU were studied. Based on these results, NCO/OH and CO/PHBD ratios of 4.0 (mol/mol) and 6/4 (g/g), respectively, were selected for the synthesis of CO/PHBD PUs using different molecular weight PHBDs. The effect of PHBD molecular weight on the resulting PUs was investigated in terms of mechanical and thermal properties. A neat CO/PU was also synthesized using the same process with an NCO/OH ratio of 4.0.

### 2.5. Analytical Procedures

#### 2.5.1. Fourier Transform Infrared

FTIR analysis was conducted using a Nicolet model Magna-IR 200 FTIR spectrometer (Thermo Fisher Scientific, Waltham, MA, USA). Dried PHBD and CO/PHBD PU samples were mixed with KBr at 1:100 ratio to make pellets. The spectra were collected in a range of 4000–400 cm^−1^ with a spectral resolution of 32 cm^−1^ and 32 scans.

#### 2.5.2. Nuclear Magnetic Resonance

^1^H-NMR measurements were recorded on a Brucker AVANCE 500 MHz spectrophotometer with deuterated chloroform as a solvent. All chemical shifts were reported in ppm relative to trimethylsilane as an internal standard.

#### 2.5.3. Molecular Weight Analysis

The number average molecular weights of the synthesized PHBDs were calculated as the ratio of ^1^H-NMR peak intensity at 4.2 ppm and 2.5 ppm as previously reported [12].

#### 2.5.4. Hydroxyl Content Determination

The hydroxyl content of the synthesized PHBD was determined by the reflux phthalation method. Briefly, 112 g phthalic anhydride was thoroughly mixed with pyridine. Then, PHBD was added to the mixture and kept at 120 °C under reflux. After the reaction, the mixture was titrated against NaOH solution using phenolphthalein as an indicator.

#### 2.5.5. Mechanical Analysis

CO/PHBD PU film samples were cut into 50 mm × 10 mm specimens and tested for their mechanical properties in terms of tensile strength and elongation at break using a universal testing machine (UTM, LR-30K, Lloyd Instruments, Hampshire, UK) with a load cell of 5 kN and a crosshead speed of 5 mm/min. Seven replicates of each film type were tested.

#### 2.5.6. Thermal Analysis

Calorimetric measurements were carried out using a Q2000 differential scanning calorimeter (DSC, TA Instruments, New Castle, DE, USA). Thermal analysis was carried out at a scanning rate of 20 °C/min. Samples (~3 mg) were heated to 200 °C and maintained for 5 min to erase previous thermal history before being cooled to −40 °C at a cooling rate of 20 °C/min. Samples were then reheated to 200 °C at a scanning rate of 20 °C/min (2nd heating) under nitrogen atmosphere using a nitrogen flow rate of 40 mL/min. Percentage crystallinity (X_c_) of the synthesized polymer samples was determined using the following Equation (1):

(1) $$X_{c}=\frac{∆H_{m}}{w∆H_{m}^{0}}\;$$

where $∆H_{m}$ is the melting enthalpy (J/g) of sample, w is the weight fraction of PHB in PU, and $∆H_{m}^{0}$ is the melting enthalpy of 100% crystalline PHB (146 J/g) [13].

Thermal degradation studies were carried out using TA Instruments D-TGA, SDT 2960 thermal analyzer. Experiments were conducted in air atmosphere at heating scans from 30 to 600 °C under dynamic heating rate of 10 °C/min (sample amount: 3–5 mg).

#### 2.5.7. Scanning Electron Microscope

The surface morphology of the synthesized PUs was analyzed using a scanning electron microscope (SEM, LEO-1530, Carl Zeiss, Zurich, Switzerland) at an accelerating voltage of 20 kV. Before analysis, film samples were placed on a SEM template, air dried, and carbon coated.

#### 2.5.8. Solubility Test

The solubility of CO/PHBD PU was measured in both chloroform and water. Films (~20 mg) were cut and placed inside vials containing 1 mL of chloroform and water. Then, the vials were continuously stirred in a shaking incubator for 24 h at room temperature. Films were then taken out of the vials and dried. Solubility was determined by the film mass loss using the following Equation (2):

(2) $$\mathrm{Solubility}\;\left(\%\right)=\frac{W_{1}-W_{2}}{W_{1\;}}\times 100$$

where W_1_ is the initial mass of the films and W_2_ is the dried mass after the test.

#### 2.5.9. Soil Biodegradation Test

Soil biodegradation test was carried out under aerobic condition. PU films cut into 15 mm × 10 mm rectangular strips were weighed (W_1_) and buried in 50 mL capacity tube containing 50 g of moist soil (pH 6.8) collected from Chungbuk National University campus. The soil height inside the tube was 100 mm and the test film samples were placed at 50 mm depth. To maintain soil humidity, 1 mL water was sprayed over the open surface of soil after 2 days. Weights of the PU films (W_d_) were measured every 7 days over a period of 35 days. After each sampling time, samples were taken out from soil, rinsed with water to remove adhered soil, and dried at room temperature for 24 h. The degree of film biodegradation was determined as the weight loss using Equation (3).

(3) $$\mathrm{Residual}\;\mathrm{weight}\;\left(\%\right)=\left(1-\frac{W_{1}-W_{d}}{W_{1}}\right)\times 100$$

## 3. Results and Discussion

### 3.1. PHB and PHBD Characterization

**Scheme 1:**
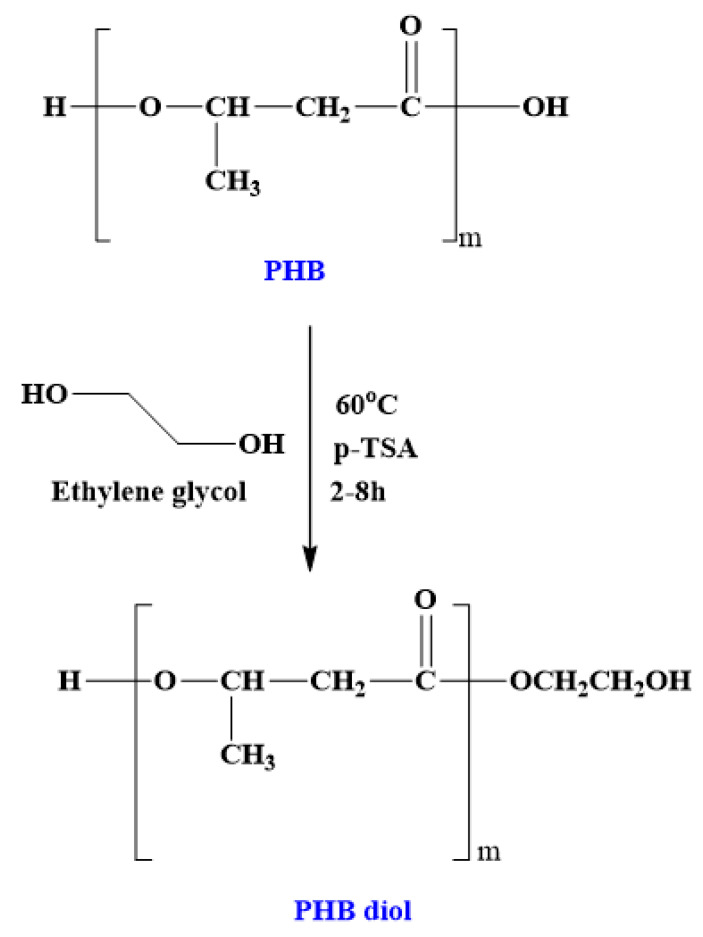
Transesterification reaction of PHB into PHB diol.

The reaction scheme related to the synthesis of PHBDs from bacterial PHB preparation is given in Scheme 1. The synthesized PHBDs were characterized in terms of FTIR, NMR, and DSC.

#### 3.1.1. FTIR Analysis 

**Figure 1:**
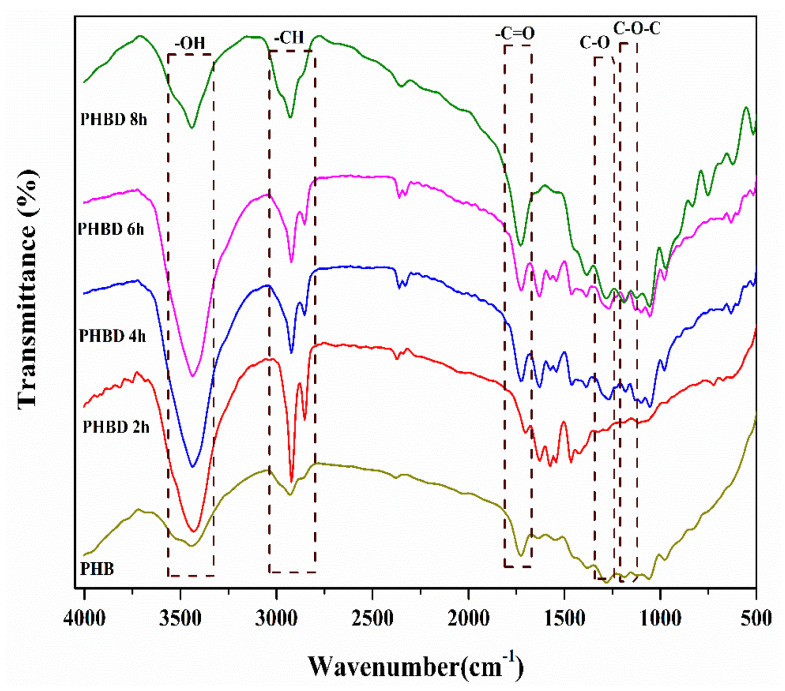
FTIR spectra of PHB and PHBDs obtained from different reaction time.

FTIR spectra of PHB and PHBDs are shown in Figure 1. Peaks at 2930 cm^−1^ and 1455–1460 cm^−1^ correspond to the –CH stretching vibrations [13]. The strong and sharp absorbance bands observed at 1710–1727 cm^−1^ are assigned to carbonyl C=O stretching modes of PHB and PHBDs [13]. The peak observed in the range of 1180–1193 cm^−1^ indicates the stretching vibration of C–O–C, while the peaks at 1250 cm^−1^ suggest the presence of the C–O stretch mode of PHB structure. The broad peak observed in PHB spectrum at 3430 cm^−1^ corresponds to hydrogen bonded –OH group with alkoxy [15]. As it can be inferred from FTIR spectra of the synthesized PHBDs, peak intensity related to hydrogen bonded –OH group increased compared to the neat PHB, suggesting the formation of hydroxyl end groups after PHB transesterification with ethylene glycol.

#### 3.1.2. ^1^H NMR Spectra

**Figure 2:**
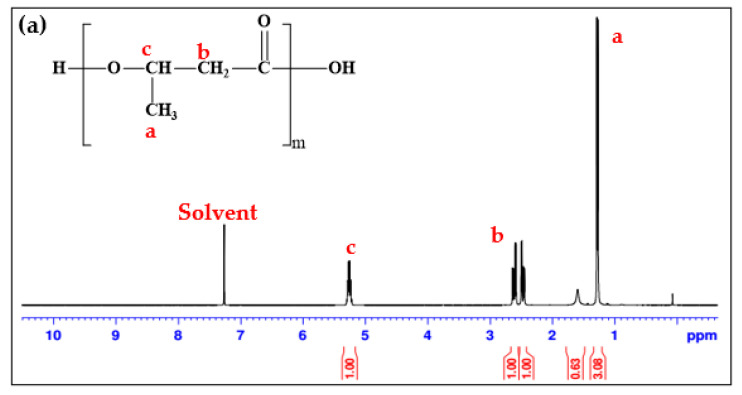

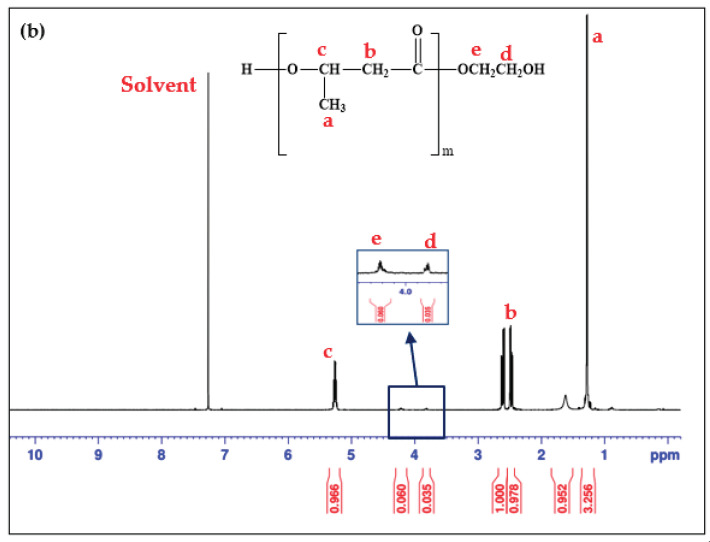
NMR spectra of (**a**) PHB and (**b**) PHBD 8h.

^1^H NMR spectra of PHB and PHBD 8h in Figure 2 display three characteristic peaks corresponding to CH_3_ group at 1.29 ppm (peak a), CH_2_ at 2.5 ppm (peak b), and CH group at 5.27 ppm (peak c), which was in accordance with the ^1^H NMR pattern reported previously [23] for PHB produced by *C. necator* KCTC 2649.

Two sets of new complex peak signals at 3.8 ppm (peak d) and 4.2 ppm (peak e) were observed, which are consistent with the formation of ester linkages between PHB terminal carboxyl group and ethylene glycol [24,25]. Furthermore, the peak area at 4.2 ppm increased with increasing reaction time denoting the successive degradation of PHB. NMR spectra of PHBD 2h to PHBD 6h are provided in Supplementary Materials (Figure S1a–c).

#### 3.1.3. Molecular Weight Analysis of Synthesized PHBD

As shown in Scheme 1, in the presence of p-TSA as a catalyst, ethylene glycol broke the ester bond and produced a double hydroxyl terminated product [26]. The molecular weights of the synthesized PHBDs are shown in Table 1. The molecular weight of PHBD drastically decreased after 2 h of alcoholysis reaction from 19233 to 7675 (g/mol) due to the random cleavage mechanism [27]. However, this decrease in molecular weight was less pronounced with increasing the reaction time [28]. In addition, the hydroxyl content analysis shows that all PHBDs contain ~2 hydroxyl groups in each structure, suggesting that the hydroxyl end-capped PHBDs were successfully synthesized.

#### 3.1.4. Thermal Properties of Synthesized PHB and PHBD

**Figure 3:**
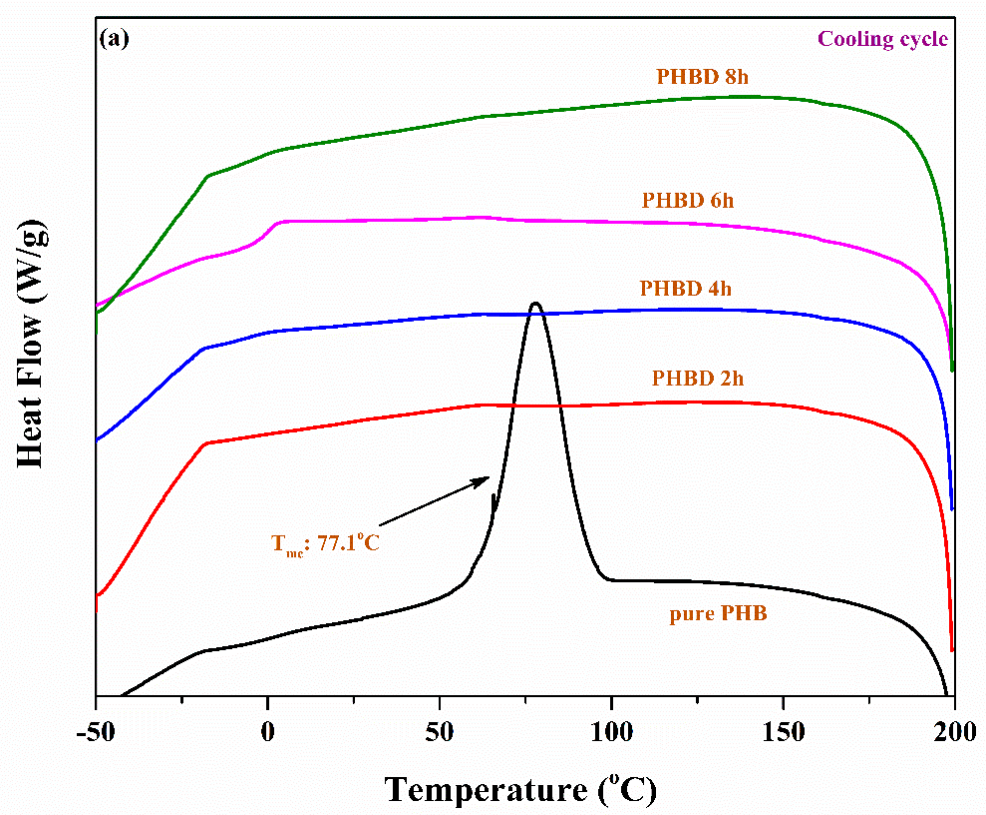

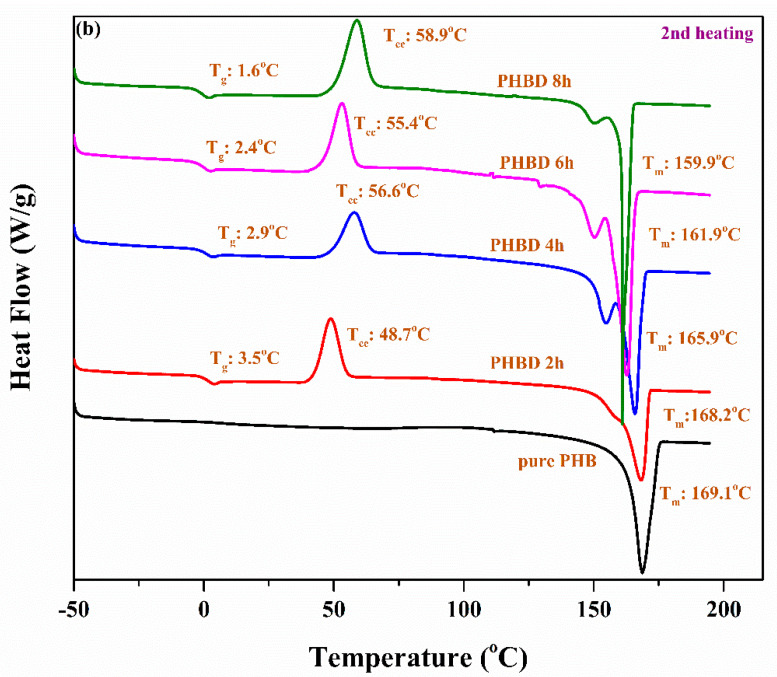
(**a**) Cooling curves and (**b**) 2nd heating curves of PHBDs.

DSC curves for the neat PHB and the synthesized PHBDs are displayed in Figure 3a,b. Thermal properties of the neat PHB and the synthesized PHBDs in terms of glass transition (T_g_), crystallization (cold crystallization (T_cc_), melt crystallization (T_mc_)), and melting temperature (T_m_) are presented in Figure 3. As shown in Figure 3a, the neat PHB polymer exhibited an exothermic melt crystallization peak (T_mc_) at 77.5 °C. However, no peak related to exothermic melting crystallization (T_mc_) was recorded for the synthesized PHBD samples. As it can be inferred from Figure 3b, all PHBD showed a cold crystallization peak (T_cc_) except neat PHB. Overall, a decrease in the glass transition values of the synthesized PHBDs was observed as a result of increasing transesterification reaction time. In this context, increasing the transesterification reaction time from 2 to 8 h led to a decrease in the T_g_ value of the corresponding PHBD from 3.5 to 1.6 °C. Similar thermal outcomes were previously observed for polypropylene glycol (PPG)-PEG-PPG and PHB diol-based PUs [29]. PHB degradation by ethylene glycol increases the mobility of PHB chains, which may decrease the glass transition of the resulting PHBDs. In addition, these synthesized polymer chains have enough mobility to crystallize during reheating scan, resulting in an enhancement of T_cc_ from 48.7 to 58.9 °C [28,29].

As shown in Figure 3b and Table 1, a slight decrease in both T_m_ and ∆H_m_ of the synthetized PHBDs was noticed compared to the neat PHB. Likewise, the crystallinity of the synthetized PHBDs decreased in the range from 43.2 to 36.4% compared to the neat PHB polymer. This reduction in crystallinity is most likely due to the decrease in the rigidity and length of the polymeric chains [29]. 

### 3.2. Structural and Morphological Characterization of CO/PHBD PU

**Scheme 2:**
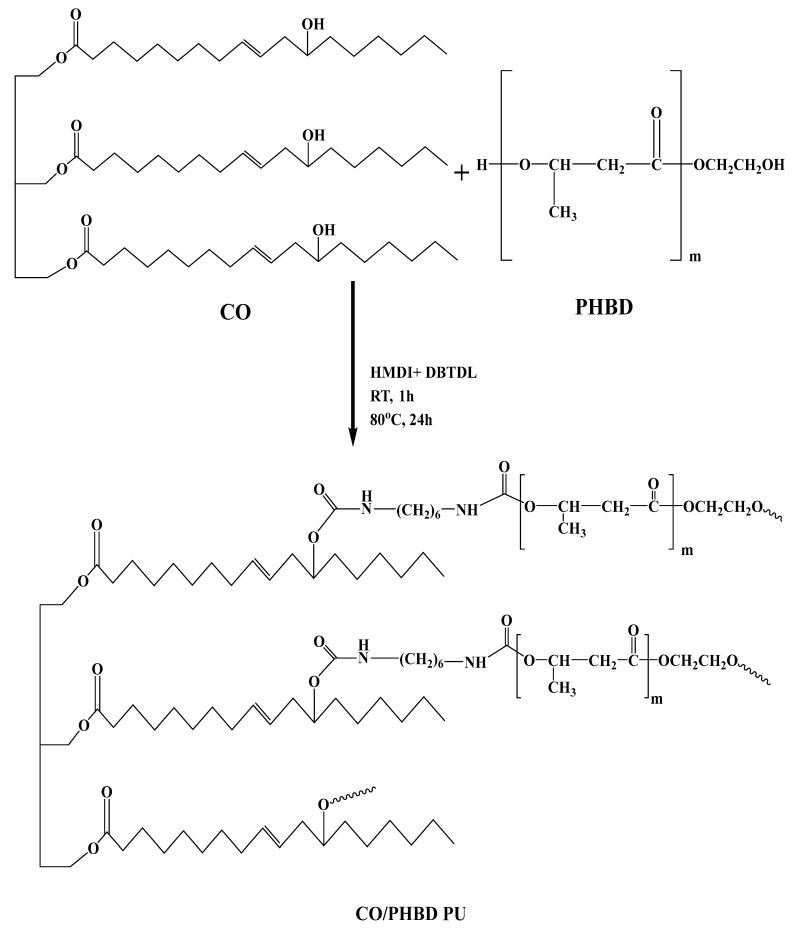
Synthetic pathway of CO/PHBD PU.

As shown in Table 1, the lowest molecular weight of PHBD was obtained after 8 h of reaction with ethylene glycol. This PHBD (PHBD 8h) was then used for a series of CO/PHBD PU formulations to investigate the effects of NCO/OH and CO/PHBD ratios. The formulations for CO/PHBD PU 8h with different CO/PHBD ratios are given in Table S1. The schematic reaction pathway of CO/PHBD PU synthesis is shown in Scheme 2. The structure of the resulting PUs was characterized in terms of FTIR and SEM.

#### 3.2.1. FTIR Spectra

**Figure 4:**
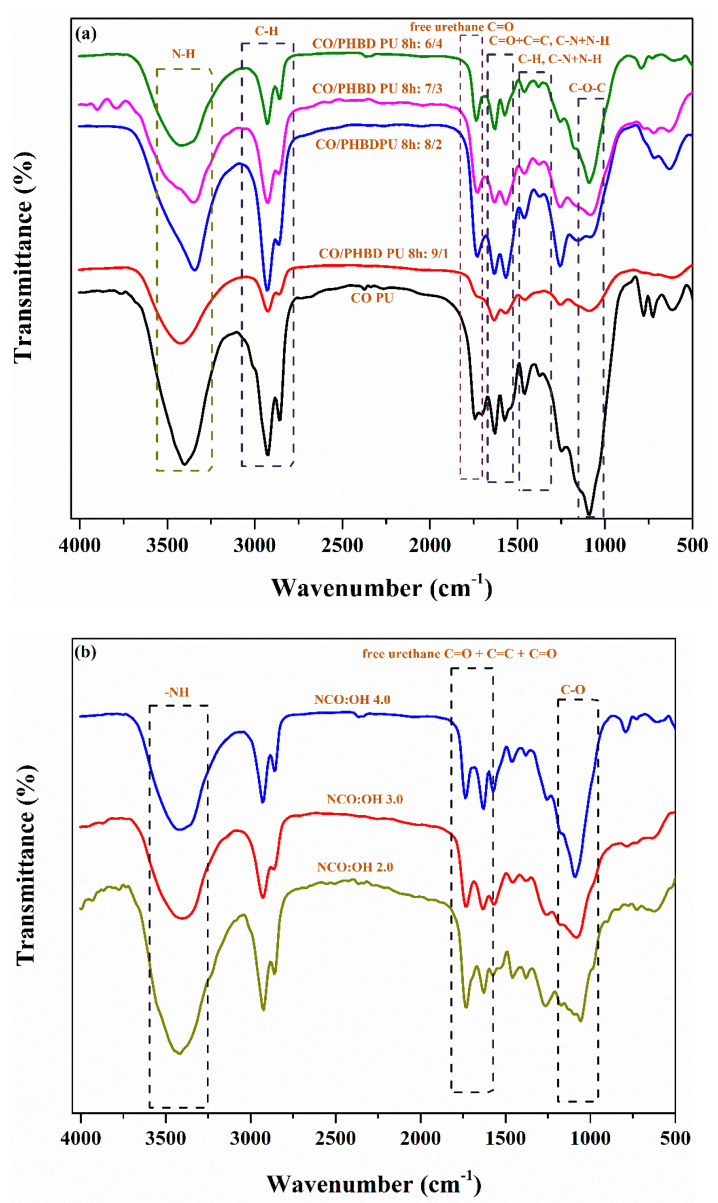

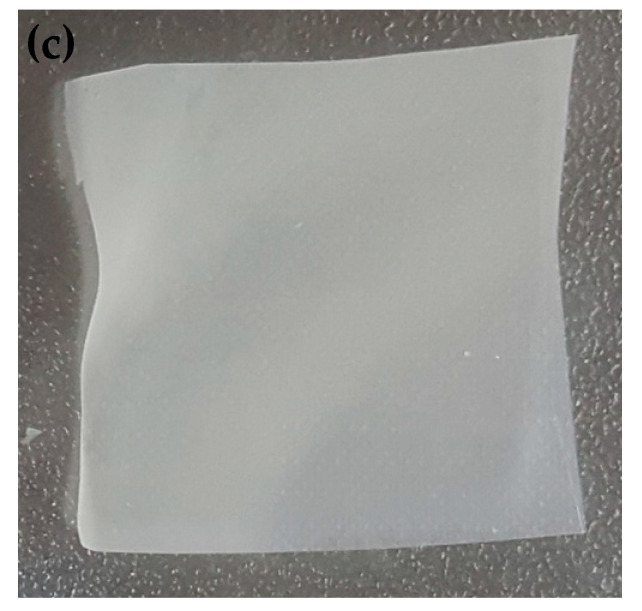
FTIR spectra of CO/PHBD PU 8h at (**a**) different CO/PHBD ratios and (**b**) different NCO/OH ratios; (**c**) image of CO/PHBD PU 8h.

FTIR spectra of the synthesized PUs at different CO/PHBDs and NCO/OH ratios are shown in Figure 4. The peaks at 3341–3416 cm^−1^ indicate the formation of N–H stretching in the PU structure [30]. Peaks at 1457 cm^−1^ and 1090 cm^–1^ confirm the presence of symmetric C–H and C–O bending vibration, respectively [29]. In addition, bands assigned to the free urethane bands (C=O) are centered around 1724–1730 cm^−1^ (amide I), while the band attributed to the formation of amide II stretching vibration (C–N and N–H) is observed in the range of 1560–1567 cm^−1^ [30]. The peaks at 1627–1632 cm^−1^ represent the double bonds (C=C) of the castor oil triglyceride chain and the formation of symmetric biuret (C=O) stretching in the PU structure [31,32]. The absence of the typical isocyanate peak at 2270 cm^−1^ suggested that excess isocyanate groups reacted with urethane to form biuret branching [32]. Moreover, the intensity of the peak at 1627–1630 cm^−1^ increased with increasing the NCO/OH ratio compared to the free urethane group (1730 cm^−1^), which may suggest the establishment of interactions between urethane and NCO groups.

#### 3.2.2. Morphological Analysis

SEM images of neat CO PU and CO/PHBD PU 8h showed smooth and cohesive surface morphology (Figure S2), suggesting the ability of short chain length PHBD to form a compatible and homogenous network with CO in the presence of HMDI. Increasing NCO/OH ratio from 2.0 to 4.0 enhanced the cohesiveness of the synthesized PUs as confirmed by the absence of holes and irregularities on the surface of PUs synthetized from the highest NCO/OH ratio of 4 (Figure S2c) [13,33].

### 3.3. Mechanical Analysis of CO/PHBD PU

#### 3.3.1. Effect of NCO/OH Ratio

**Figure 5:**
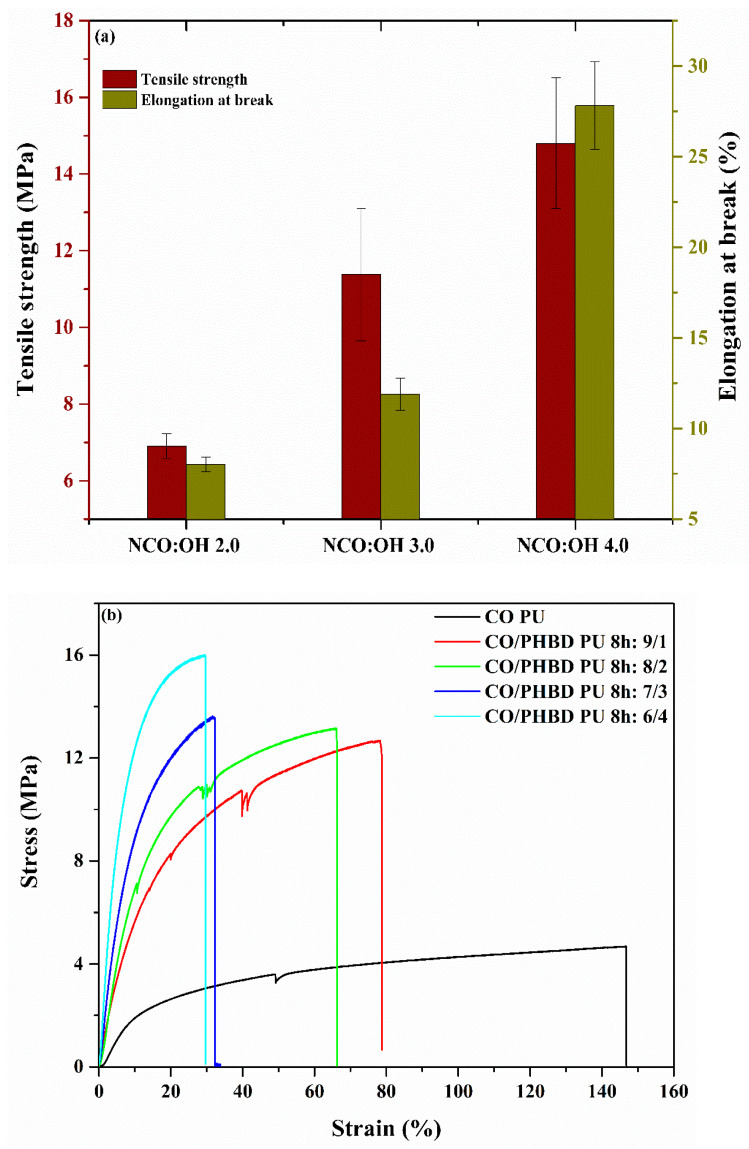
(**a**) Effect of NCO/OH ratio (CO/PHBD ratio: 6/4) and (**b**) CO/PHBD ratio (NCO/OH ratio: 4.0) on mechanical properties of the resulting PU.

The effect of NCO/OH ratio on the mechanical properties of CO/PHBD PU 8h is shown in Figure 5a. It was observed that the CO/PHBD PU at an NCO/OH ratio of 1.0 was too weak to be mechanically tested. Increasing NCO/OH ratio from 2.0 to 4.0 significantly increased the tensile strength and elongation at break of the resulting CO/PHBD PU from 7.16 to 14.8 MPa and from 7.70 to 27.4%, respectively. This trend was in agreement with previous studies [33,34]. Such improvement in the mechanical properties of the resulting CO/PHBD PU can be attributed to the ability of HMDI to enhance the interfacial adhesion between CO and PHBD, leading to the formation of a compatible and homogenous network as revealed by SEM analysis [34,35,36].

#### 3.3.2. Effect of CO/PHBD Ratio

The stress-strain curves of the synthesized PUs at different CO/PHBD ratios using a constant NCO/OH ratio of 4.0 is shown in Figure 5b. Overall, increasing PHBD content significantly increased the tensile strength of the resulting CO/PHBD PU. This increase was estimated by 300% for CO/PHBD PU incorporating the highest PHBD content (6/4 CO/PHBD PU) compared with the neat CO PU. However, a significant decrease in the elongation at break of the resulting CO/PHBD PU was recorded as a result of increasing PHBD content. This behaviour can be ascribed to the formation of biurets inside the PU matrix [37] along with the high crystallinity of the incorporated PHB. Nonetheless, the resulting CO/PHBD PU becomes brittle with further increase in PHBD content. As shown in DSC analysis (discussed later), the CO PU did not reveal any crystalline peak which confirms the amorphous structure of the resulting film [37]. Upon the addition of PHBD, crystalline peaks were observed denoting the semicrystalline structure of the synthesized PHBD, which may explain the high stiffness of the resulting CO/PHBD PU. Such increase in tensile strength may restrict the molecular chain movement in polymers and ultimately decreased their elongation at break. Further, with increasing PHBD content, the portion of castor oil as soft segment decreased, which may explain in part the decrease in the flexibility of the resulting CO/PHBD PU. Overall, CO/PHBD PU 8h produced in this study exhibited higher tensile strength compared to CO/cellulose-based PU [38], CO-derived poly(urethane urea) [19], and CO/MDI-modified cellulose acetate-based PU [21]. However, the elongation at break of the synthesized CO/PHBD PU 8h was lower than that of the CO-based PUs reported in the studies mentioned above.

### 3.4. Thermal Analysis of CO/PHBD PU

#### 3.4.1. Differential Scanning Calorimetry of CO/PHBD PU

**Figure 6:**
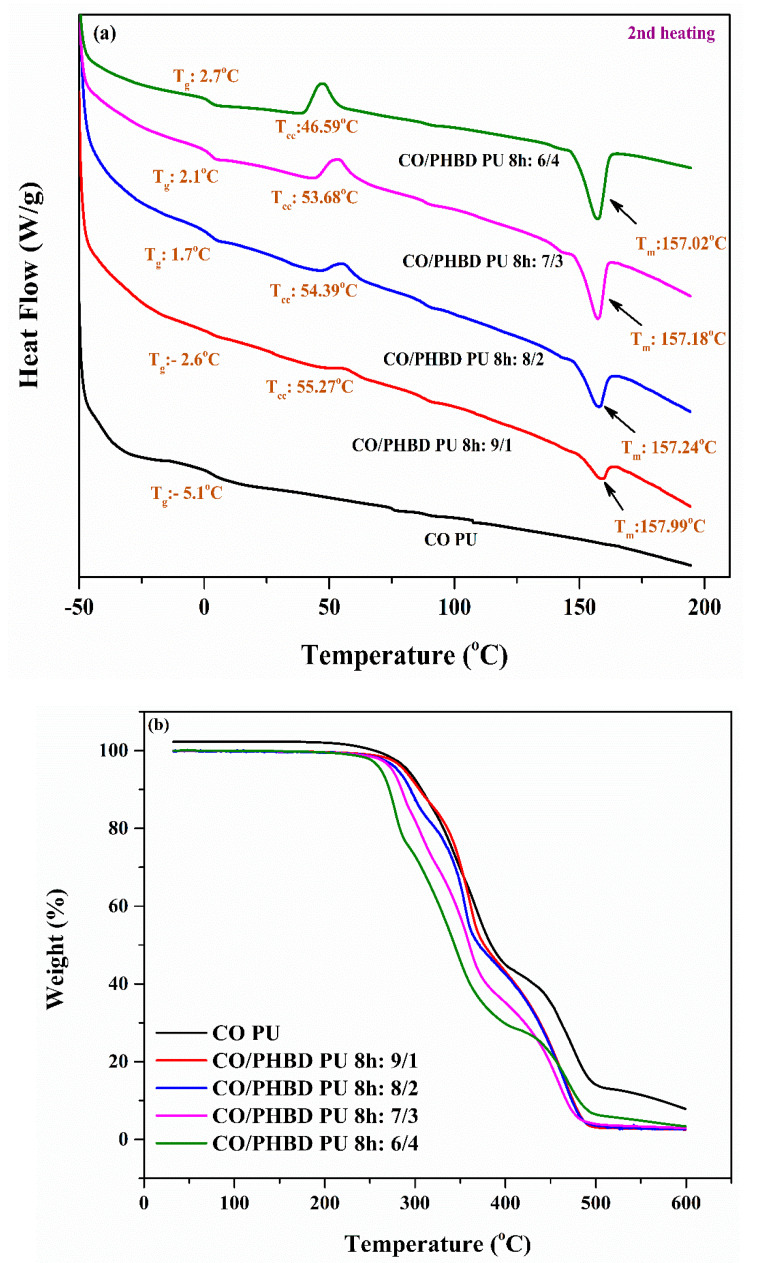

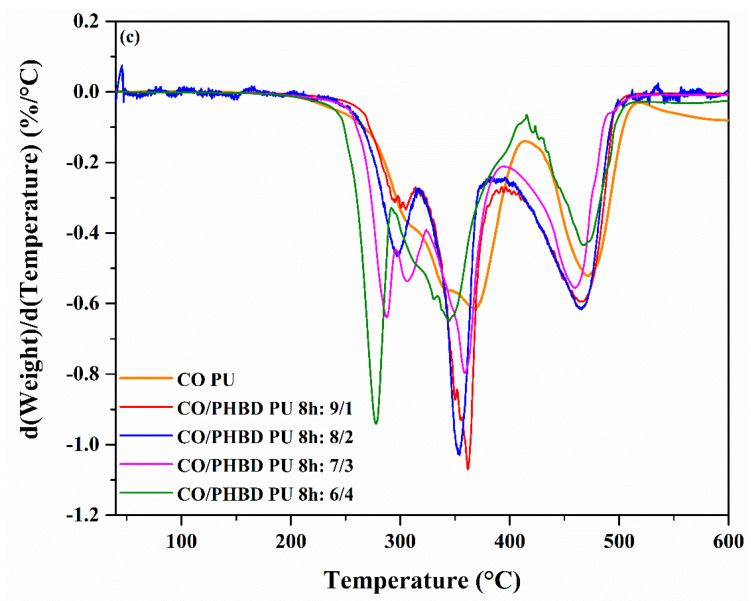
(**a**) DSC, (**b**) TGA, and (**c**) derivative TGA curves of CO/PHBD PU 8h.

The second heating curves of the synthesized CO/PHBD PU 8h at different PHBD contents are shown in Figure 6a. Regardless of the CO/PHBD ratio, only one T_g_ was detected at the range of −5.1 to 2.7 °C [9,12]. Overall, T_g_ increased upon the incorporation of PHB which may suggest a partial mixing between hard and soft segment phase [15]. Neither crystallization nor melting peaks were detected for the pure CO PU confirming the amorphous structure of the polymer. As shown in Figure 6a, crystallization and melting peaks appeared in the thermograms of the synthesized PUs upon the incorporation of PHBD. A slight shift in cold crystallization temperature (T_cc_) to lower values was recorded as a result of increasing PHBD content, indicating an increase in the crystallization rate of PHBD component from the glassy state in the synthesized PUs [9]. This behavior can be attributed to the decrease in CO content which may decrease the chemical crosslinking degree in the PU matrix. Melting enthalpies of CO/PHBD PU 8h significantly increased from 1.00 to 8.13 J/g with increasing PHBD content. No evident shifts occurred in the melting peak of the synthesized PUs as a result of increasing PHBD content. Overall, the melting enthalpies of the synthesized PUs were lower than the pure PHBD (∆H_m_ 63.1 J/g), which might be resulted from the disruption in PHB chain by soft CO dangling structure and phase mixing between CO and PHBD segments [9,36]. In fact, this phase mixing is expected to reduce the homogeneity of PHB chains. As shown in Table 2, increasing PHBD content significantly increased the percentage crystallinity of the synthesized CO/PHBD PU from 6.87 to 14.0%.

#### 3.4.2. Thermogravimetric Analysis of CO/PHBD PU

TGA and derivative TGA (DTGA) curves of the synthesized PUs are shown in Figure 6b,c. As it can be inferred from the figures, both neat CO PU and CO/PHBD PU 8h showed a similar pattern of thermal behavior with three thermal degradation steps. For neat CO PU, the first degradation step at 278–302 °C corresponds to thermally labile urethane linkages, whereas the second and third steps are due to the degradation of CO triglyceride structure [9,37]. As shown in Figure 6c, new evident peaks at 270–300 °C appeared in DTGA curves of CO/PHBD PU 8h, which are associated with the degradation of PHB segment as a consequence of its low thermal stability, while the second and third steps correspond to CO decomposition [9]. Urethane dissociation produces isocyanate, alcohol, primary amines, and olefins through the formation of secondary amines and CO_2_ [39].

TGA data showed that T_10%_ (temperature at 10% loss) and T_50%_ decreased with increasing PHBD content (Table 2), indicating a decrease in the thermal stability of the resulting CO/PHBD PU 8h most likely due to the reduction in stable triglyceride molecule.

### 3.5. Solubility of CO/PHBD PU

The solubility of the synthesized CO/PHBD PU was determined in water and chloroform. As shown in Figure S3, the film solubility in chloroform (~15%) was significantly higher than in water (~2%). In addition, the solubility of the films increased with increasing PHBD content due to the high solubility of PHBD in chloroform (42 g/L). However, the synthesized films are moderately resistant to organic solvent as the loss in the weight of the films was relatively low (<15%).

### 3.6. Effect of Different Molecular Weight PHBD

The different molecular weight PHBDs obtained after 2 to 8 h were used for the synthesis of their corresponding PUs. These different PUs were formulated at a constant NCO/OH ratio of 4.0 and a CO/PHBD ratio of 6/4, respectively. The effect of the different molecular weight PHBDs on the structural and physical properties of the resulting CO/PHBD PU was evaluated. Likewise, the biodegradability of the different CO/PHBD PU was evaluated.

#### 3.6.1. Structural Analysis

The FTIR spectra of the PUs resulting from the different timed reaction PHBDs did not reveal any differences between the different synthesized PUs (Figure S4). All PU spectra displayed N–H stretching, free urethane, amide, and C–O–C groups at 3405–3410 cm^−1^, 1731–1733 cm^−1^, 1623–1627 cm^−1^, and 1052–1096 cm^−1^, respectively. The NCO peak at 2270 cm^−1^ completely disappeared for all the synthesized PUs, suggesting the formation of biuret linkage.

**Figure 7:**
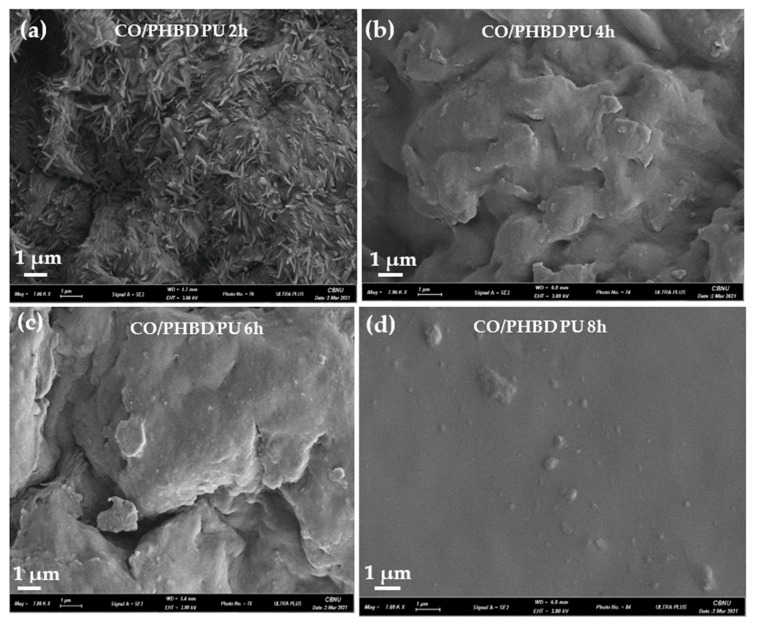
Morphological images of (**a**) CO-PHBD PU 2h, (**b**) CO/PHBD PU 4h, (**c**) CO/PHBD PU 6h, and (**d**) CO/PHBD PU 8h.

The SEM images of the synthesized PUs are shown in Figure 7. Overall, PUs produced from higher molecular weight PHBDs showed rough and heterogeneous surfaces, most likely due to the low compatibility between CO and the incorporated higher molecular weight PHBDs [39]. In fact, longer molecular chains of PHBD may cause steric hindrance, leading to the poor miscibility between CO and PHBD.

#### 3.6.2. Mechanical and Thermal Analysis

**Figure 8:**
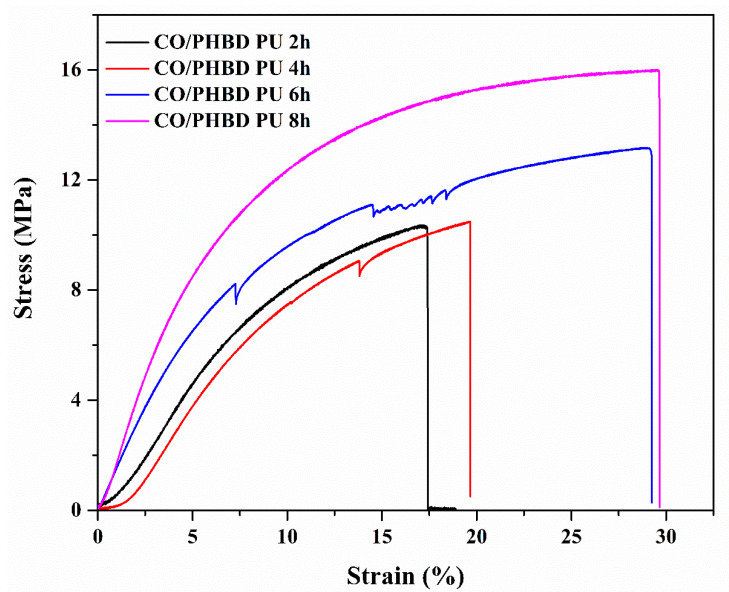
Stress vs. strain curves of the different CO/PHBD PUs.

The stress–strain curves of different CO/PHBD PUs are shown in Figure 8. Tensile properties and thermogravimetric data of CO/PHBD PUs are summarized in Table 3. Both tensile strength and elongation at break increased with decreasing the molecular weight of the synthesized PHBDs. Overall, tensile strength increased from 10.8 MPa for CO/PHBD PU 2h to 15.3 MPa for CO/PHBD PU 8h. Simultaneously, elongation at break increased from 17.2% to 28.8%. As revealed by SEM analysis, such decrease in the mechanical properties with increasing molecular weight of PHBD can be attributed to the increase in the surface heterogeneity and roughness of the corresponding CO/PHBD PUs (Figure 7a–c), most likely due to the low reactivity between CO and high molecular weight PHBDs. Longer molecular chains of PHBD might cause steric hindrance in the PU matrix [39].

**Figure 9:**
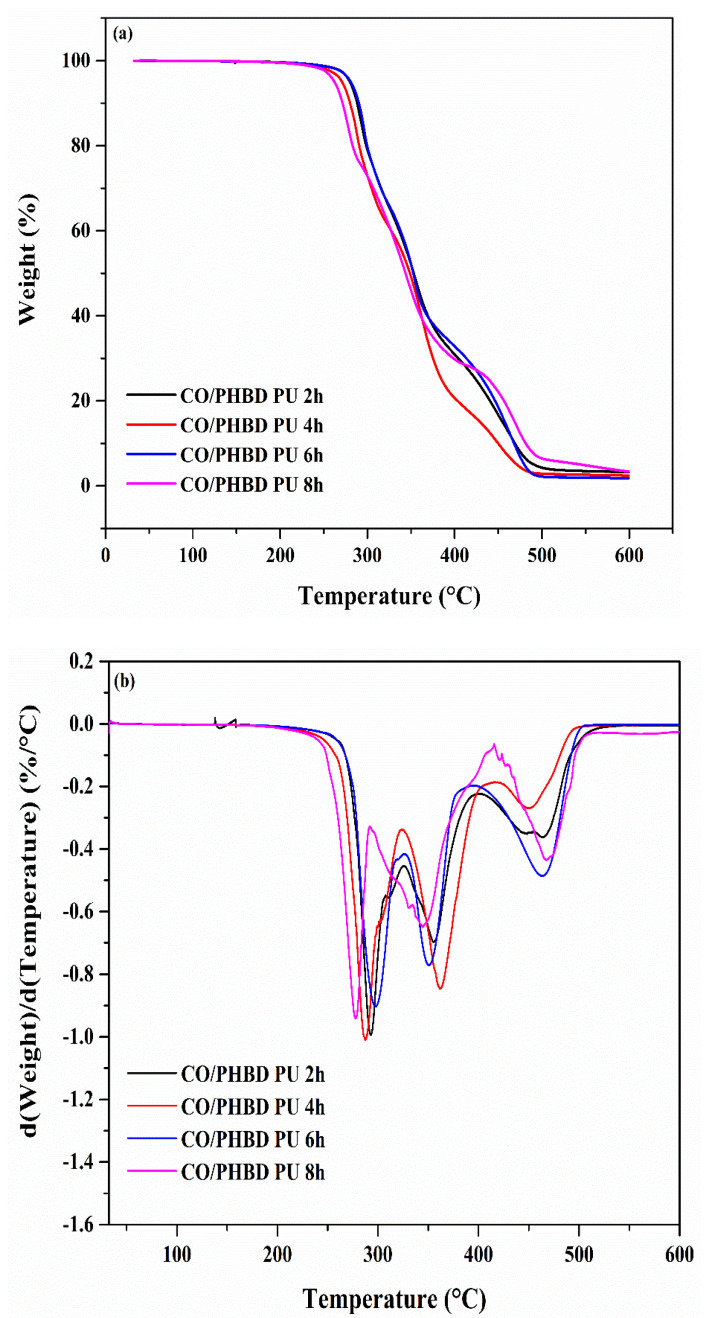
(**a**) TGA and (**b**) derivative TGA curves of CO/PHBD PUs.

TGA and DTGA curves of the synthesized PUs are shown in Figure 9. All of the synthesized PUs exhibit three thermal degradation steps. As it can be inferred from Table 3, PUs resulting from high molecular weight PHBDs showed higher T_10%_ and T_50%_ values compared to those synthesized from short chain PHBDs [40,41]. It is assumed that increasing PHBD molecular weight decreased the concentration of the thermally labile urethane concentration which may explain the observed increase in the thermal stability of the corresponding PUs.

#### 3.6.3. Soil Biodegradation Studies

**Figure 10:**
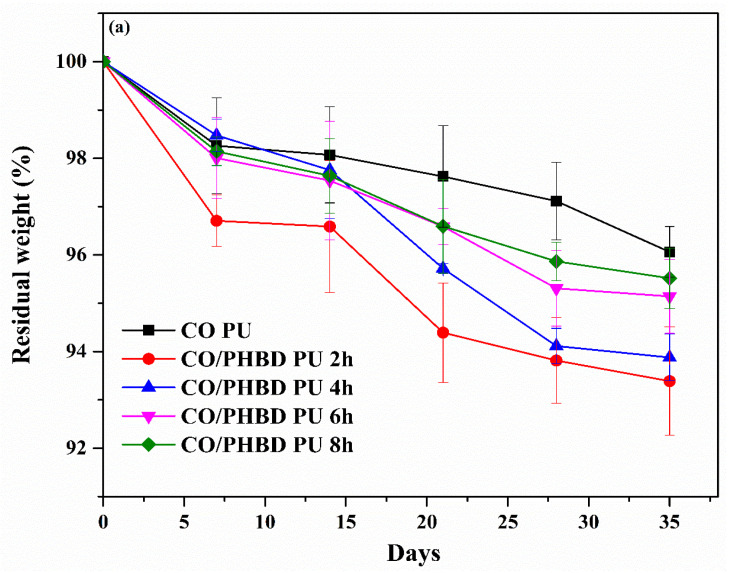

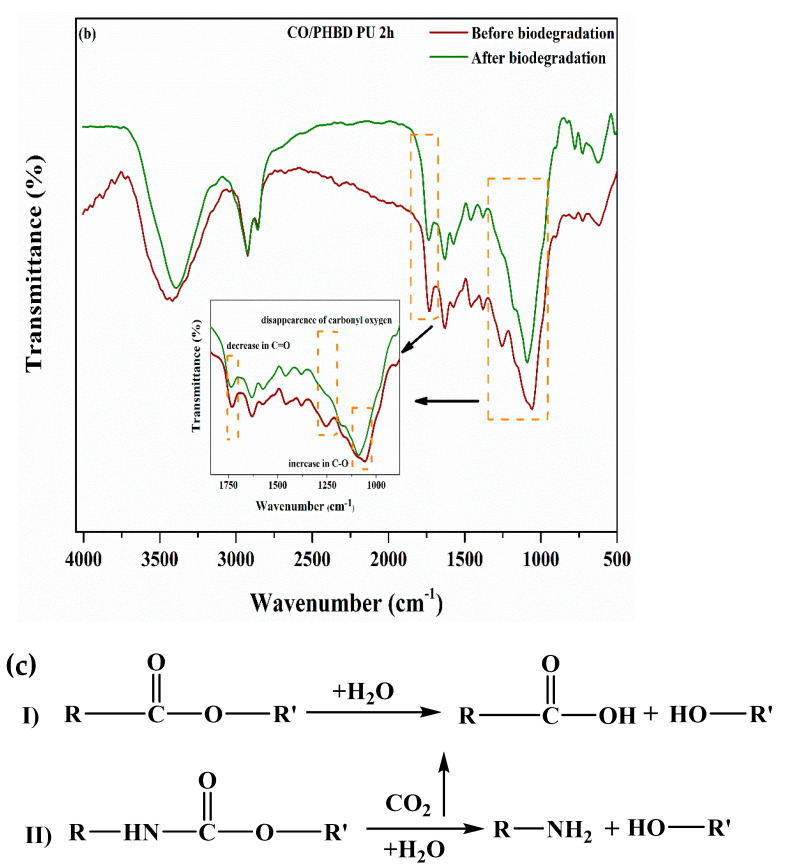
**(a**) Residual weight with time, (**b**) FTIR analysis of CO/PHBD PU after soil biodegradation (after 35 days), and (**c**) a possible mechanism for the biodegradation process of CO/PHBD PU.

The residual weights of CO/PHBD PUs synthesized from the different molecular weight PHBDs are presented in Figure 10a. Overall, both neat CO PU and CO/PHBD PUs exhibited low biodegradation rate in soil after 35 days. This low biodegradation ability can be attributed to the high hydrophobicity of CO chains along with the high crystallinity of the incorporated PHBDs [11,13]. As it can be inferred from Figure 10a, the biodegradation rate of CO/PHBD PU 2h was slightly higher (residual weight ~ 93.0%) compared to CO/PHBD PU 8h (residual weight ~ 95.5%) and the neat CO PU (residual weight ~ 96.1%). Our findings were consistent with previous reports concerning the biodegradation of CO-based PUs [13,42]. The relatively higher biodegradation rate recorded for CO/PHBD PU 2h can be ascribed to the presence of higher number of repetitive units in the PHB molecule. Regardless of the different molecular weight PHBDs, the weight loss of the different CO/PHBD PU increased rapidly during the first 7 days and then slowed down. This rapid degradation step can be attributed to the hydrolysis of the PHB ester bonds, while the slow degrading step can be ascribed to the hydrolysis of urethane group [13]. On the other hand, structural changes caused by soil biodegradation during the burial test was evaluated by FTIR (Figure 10b). The FTIR spectrum of CO/PHBD PU 2h showed a slight decrease in the intensity of peaks at 1730 cm^−1^ and an increase in the intensity of the peaks at 1080–1090 cm^−1^ after soil biodegradation, denoting the hydrolysis of urethane group and the formation of alcohol C–O group [12]. In addition, the absorption peak at 1260 cm^−1^ disappeared after 35 days of soil burial which is related to the degradation of carbonyl oxygen linkage of the ester groups located on the PU surface [11,43]. Moreover, a slight increase in the intensity of the peak corresponding to N–H stretching at 1567 cm^−1^ was noticed denoting the deformation of urethane bonds [44]. A possible mechanism for the biodegradation process of CO/PHBD PU is presented in Figure 10c [13]. Further, small pores, cracks, and grooves appeared on the PU surface after 35 days of biodegradation which may suggest the ability of water and microorganism present in the soil to diffuse inside the PU matrix (Figure S5). Such increase in erosion of the PU surface may be considered as a good indicator of the biodegradation process of CO/PHBD PUs [45]. The resulting CO/PHBD PUs may have potential in biomedical, pharmaceutical, packaging, and agriculture fields due to their biodegradability and improved mechanical properties over pure CO-PU or PHB [16,44,45,46,47].

## 4. Conclusions

In this study, biodegradable biobased PUs were successfully produced from CO and PHBD using HMDI as a crosslinking agent. First, a series of PHBDs with different molecular weights were synthesized through transesterification of bacterial PHB and ethylene glycol by changing the reaction time. The synthesized PHBDs were characterized by FTIR, NMR, and DSC. The structural, mechanical, and thermal properties of CO/PHBD PUs were measured by varying the NCO/OH molar ratio and CO/PHBD content. Our results revealed an increase by approximately 300% in the tensile strength of CO/PHBD PU produced at a CO/PHBD weight ratio of 6/4 and an NCO/OH molar ratio of 4.0 compared to neat CO PU. The glass transition temperature and crystallinity of PU significantly increased with increasing the PHBD content. In addition, CO/PHBD PUs synthetized from short chain PHBD exhibited higher tensile strength compared to those produced from long chain PHBD, due to the good compatibility between CO and the corresponding PHBD. Likewise, the highest biodegradation rate was recorded for CO/PHBD PU synthetized from short chain PHBD probably due to the presence of a high number of repetitive groups in the PHB molecule.

## Figures and Tables

**Table 1:** Molecular weight of PHBDs at different reaction time.

Reaction Time (h)	M_n_ of PHBD (g/mol)	∆H_m_ (J/g)	Hydroxyl Content (mol)
0	-	63.1	-
2	19233 ± 20	55.7	1.99
4	7675 ± 16	54.8	1.98
6	4817 ± 18	53.9	1.96
8	1890 ± 16	53.2	1.92

**Table 2:** Crystallinity, melting enthalpy, and TGA data of CO/PHBD PU 8h.

CO/PHBD	∆H_m_ (J/g)	X_c_ (%)	T_10%_ (°C)	T_50%_ (°C)	T_90%_ (°C)
1/0	-	-	301	379	534
9/1	1.00	6.87	305	377	475
8/2	2.89	9.91	295	371	474
7/3	4.65	10.6	285	361	468
6/4	8.13	14.0	272	344	481

**Table 3:** Mechanical and thermal data for CO/PHBD PUs.

Sample	Tensile Strength (MPa)	Elongation at Break (%E)	T_10%_ (°C)	T_50%_ (°C)	T_90%_ (°C)
CO/PHBD PU 2h	10.8 ± 0.9	17.2 ± 0.8	288	353	446
CO/PHBD PU 4h	11.3 ± 1.2	18.1 ± 1.5	280	351	450
CO/PHBD PU 6h	12.1 ± 1.0	27.2 ± 1.8	275	348	468
CO/PHBD PU 8h	15.3 ± 1.0	28.8 ± 2.4	272	344	481

## Data Availability

Data available on request.

## Supplementary Materials

The following are available online at https://www.mdpi.com/article/10.3390/polym13091387/s1. Table S1: CO/PHBD PU 8h formulations at different CO/PHBD ratio; Figure S1: ^1^HNMR spectra of PHB diols obtained from different timed reactions; Figure S2: SEM images of CO PU (a) and CO/PHBD PU 8h with NCO/OH 2.0 (b) and 4.0 (c); Figure S3: Solubility test of CO/PHBD PUs in water and chloroform; Figure S4: FTIR spectra of different CO/PHBD PUs; Figure S5: SEM analysis of CO/PHBD PUs after 35 days soil burial test.
