# Supplementary material for: Biodegradable Polyurethanes Based on Castor Oil and Poly (3-hydroxybutyrate)
Source: Polymers (Basel). 2021 Apr 24;13(9):1387. doi: 10.3390/polym13091387 (PMC8123115; doi:10.3390/polym13091387)
Supplement: Supplementary file 1 [file polymers-13-01387-s001.zip › polymers-1185972-supplementary.pdf]

polymers

Article

Biodegradable Polyurethanes Based on Castor Oil and Poly (3-hydroxybutyrate)

Pathikrit Saha, Chanin Khomlaem, Hajer Aloui and Beom Soo Kim *

Department of Chemical Engineering, Chungbuk National University, Cheongju, Chungbuk 28644, Korea; pathikritsaha89@gmail.com (P.S.); nament99@gmail.com (C.K.); hajer.aloui@ymail.com (H.A.)
* Correspondence: bskim@chungbuk,ac,kr; Tel.: +82 43 261 2372

Citation: Saha, P.; Khomlaem, C.; Aloui, H.; Kim, B.S. Biodegradable Polyurethanes Based on Castor Oil and Poly (3-hydroxybutyrate). *Polymers* **2021**, *13*, 1387. https://doi.org/10.3390/polym13091387

Academic Editor: Arantxa Eceiza Mendiguren

Received: 31 March 2021
Accepted: 21 April 2021
Published: 24 April 2021

Publisher's Note: MDPI stays neutral with regard to jurisdictional claims in published maps and institutional affiliations.

Copyright: © 2021 by the authors. Licensee MDPI, Basel, Switzerland. This article is an open access article distributed under the terms and conditions of the Creative Commons Attribution (CC BY) license (http://creativecommons.org/licenses/by/4.0/).(http://creativecommons.org/licenses/by/4.0/).

Table S1. CO/PHBD PU 8h formulations at different CO/PHBD ratio.

CO/PHBD (g/g)*	Mass of CO (g)	OH molar equivalent in CO	Mass of PHBD (g)	OH molar equivalent in PHBD**
1/0	1	0.0032	-	-
9/1	0.9	0.0029	0.1	0.0001
8/2	0.8	0.0026	0.2	0.0002
7/3	0.7	0.0023	0.3	0.0003
6/4	0.6	0.0019	0.4	0.0004

* Total mass: 1 g.

** OH functionality in PHBD 8h: 1.92.

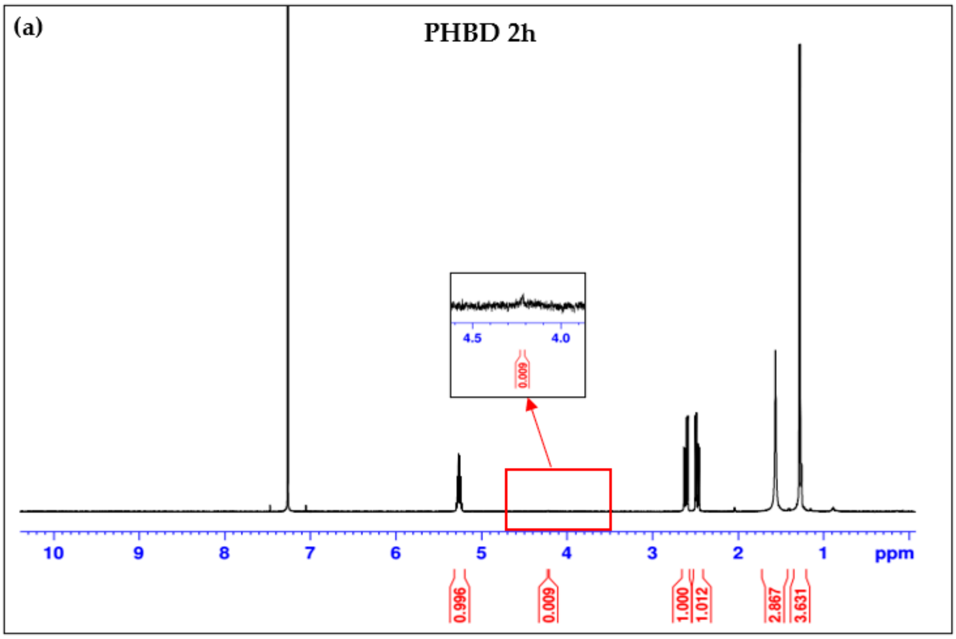

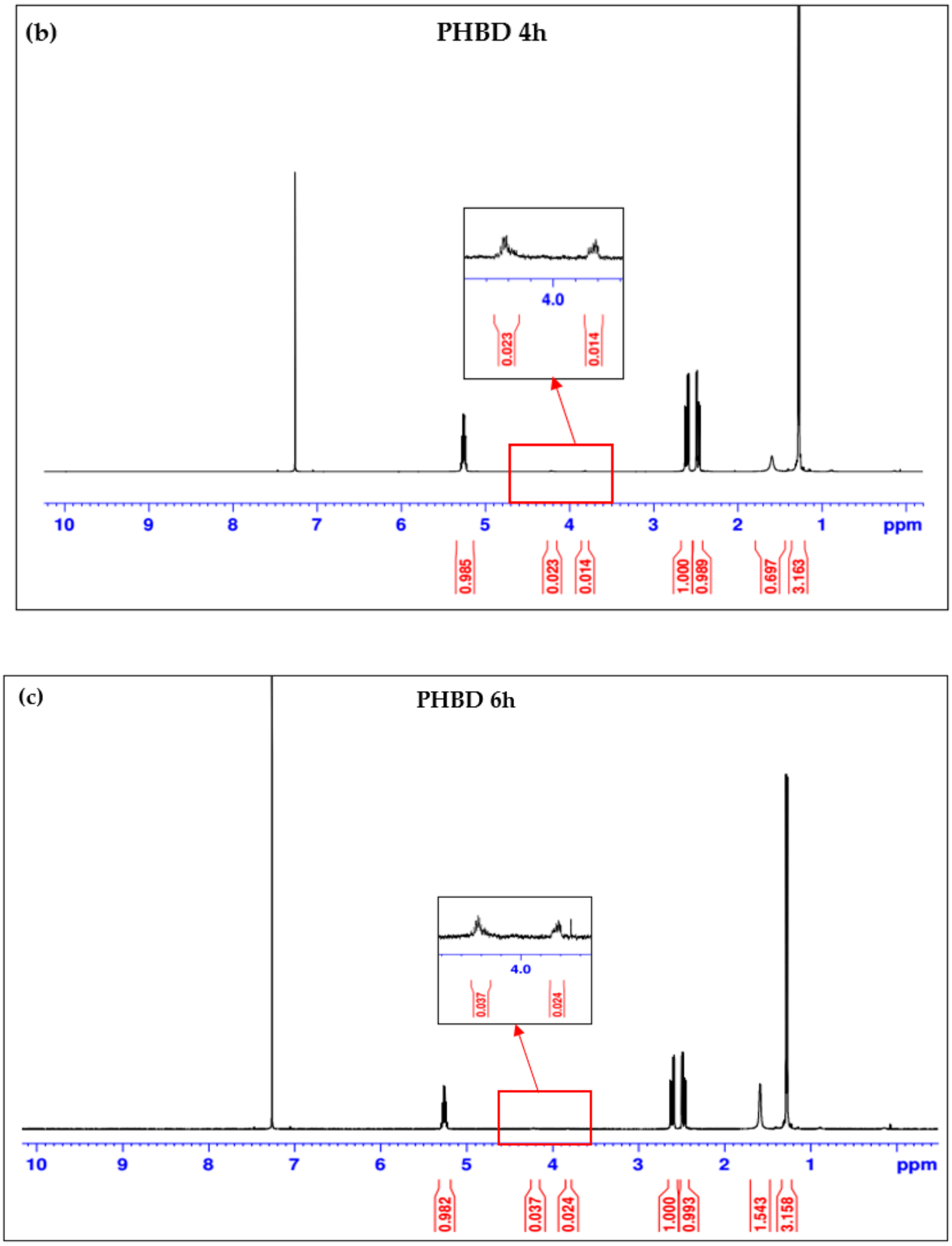

Figure S1. ^{1}HNMR spectra of PHB diols obtained from different timed reactions.

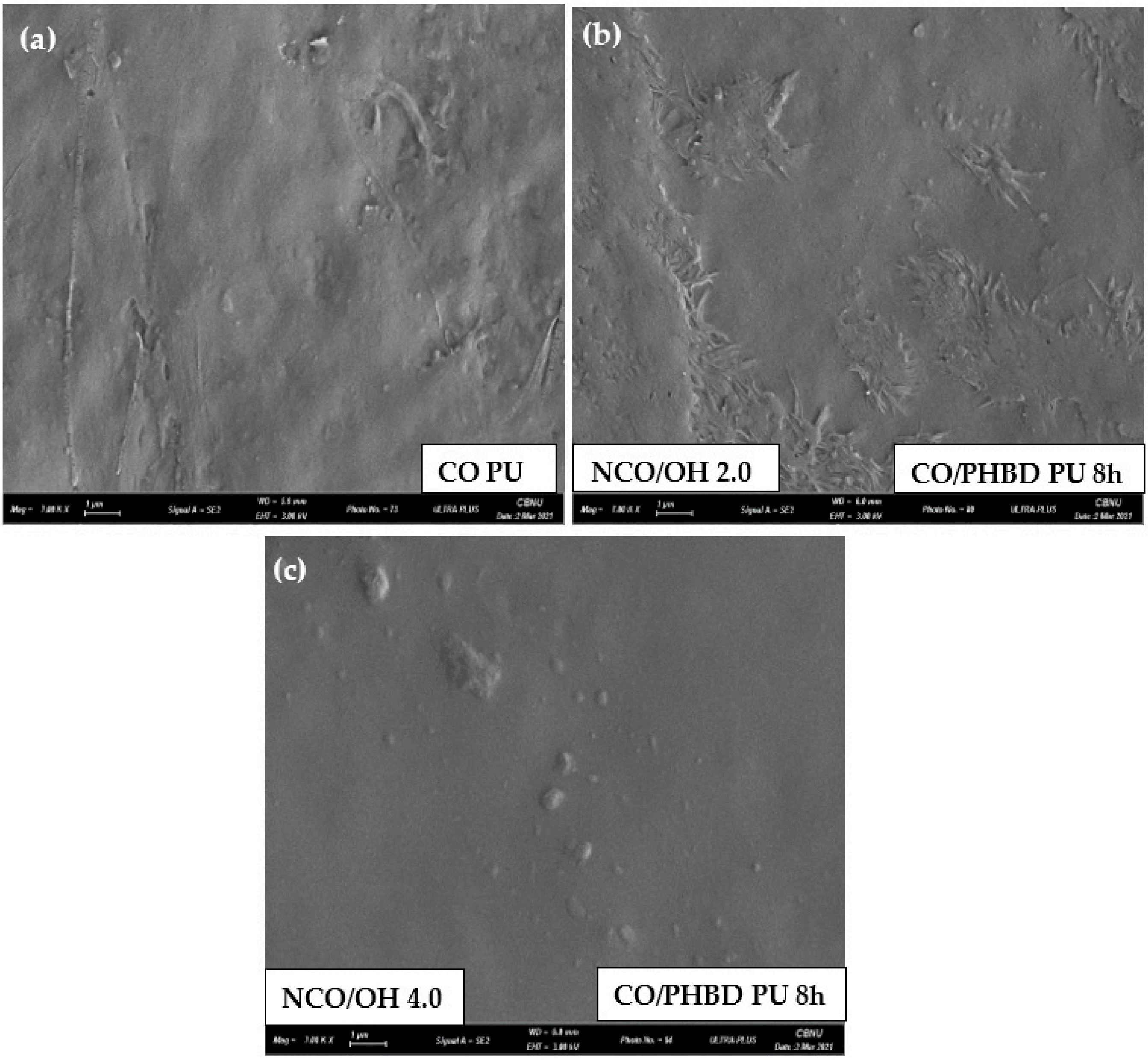

Figure S2. SEM images of CO PU (**a**) and CO/PHBD PU 8h with NCO/OH 2.0 (**b**) and 4.0 (**c**).

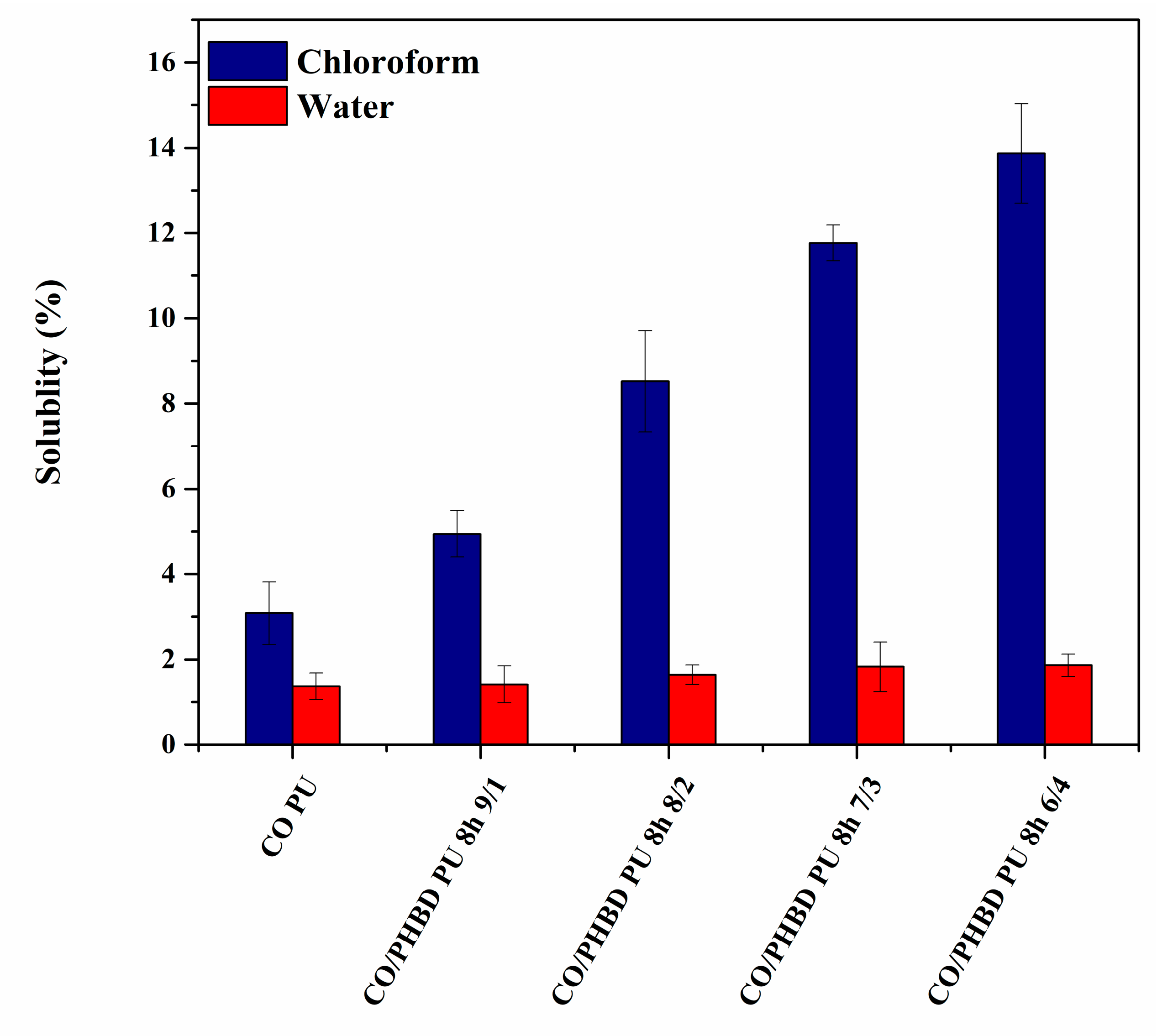

Figure S3. Solubility test of CO/PHBD PUs in water and chloroform.

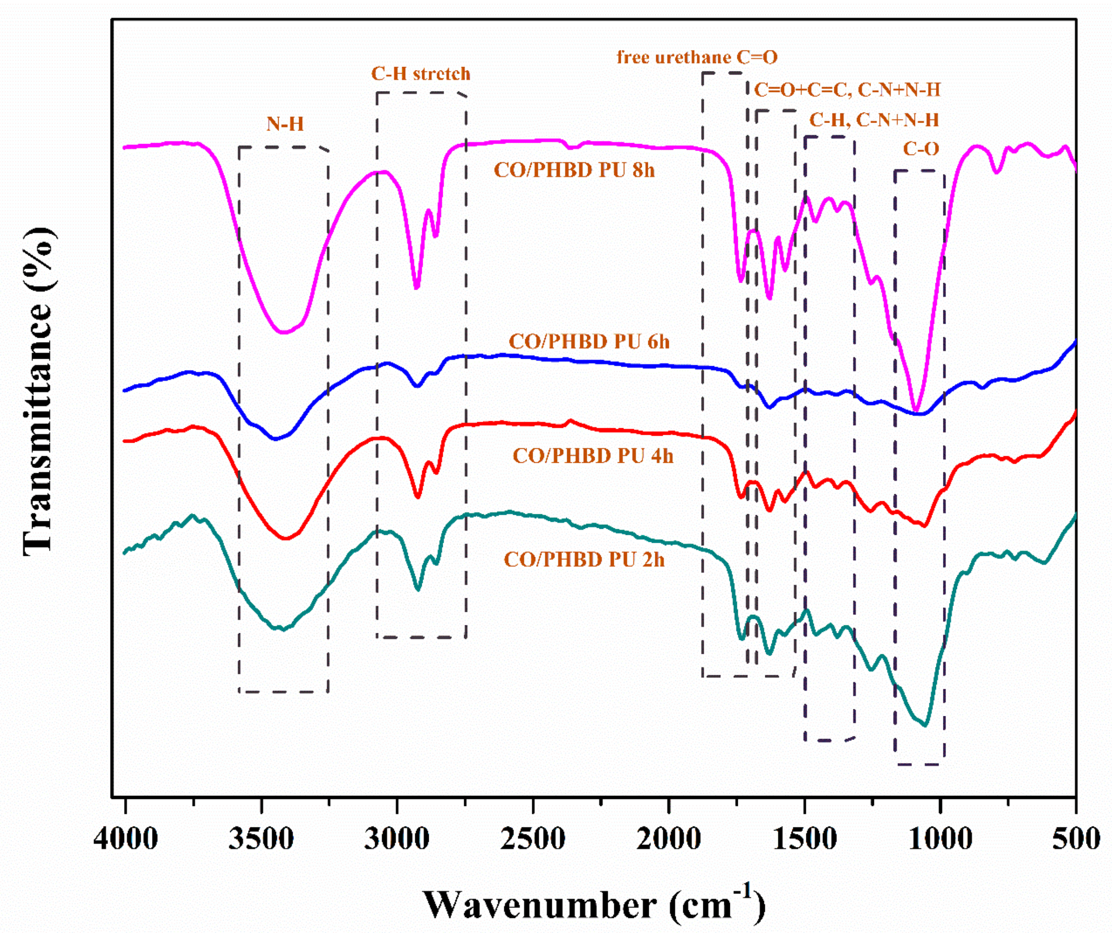

Figure S4. FTIR spectra of different CO/PHBD PUs.

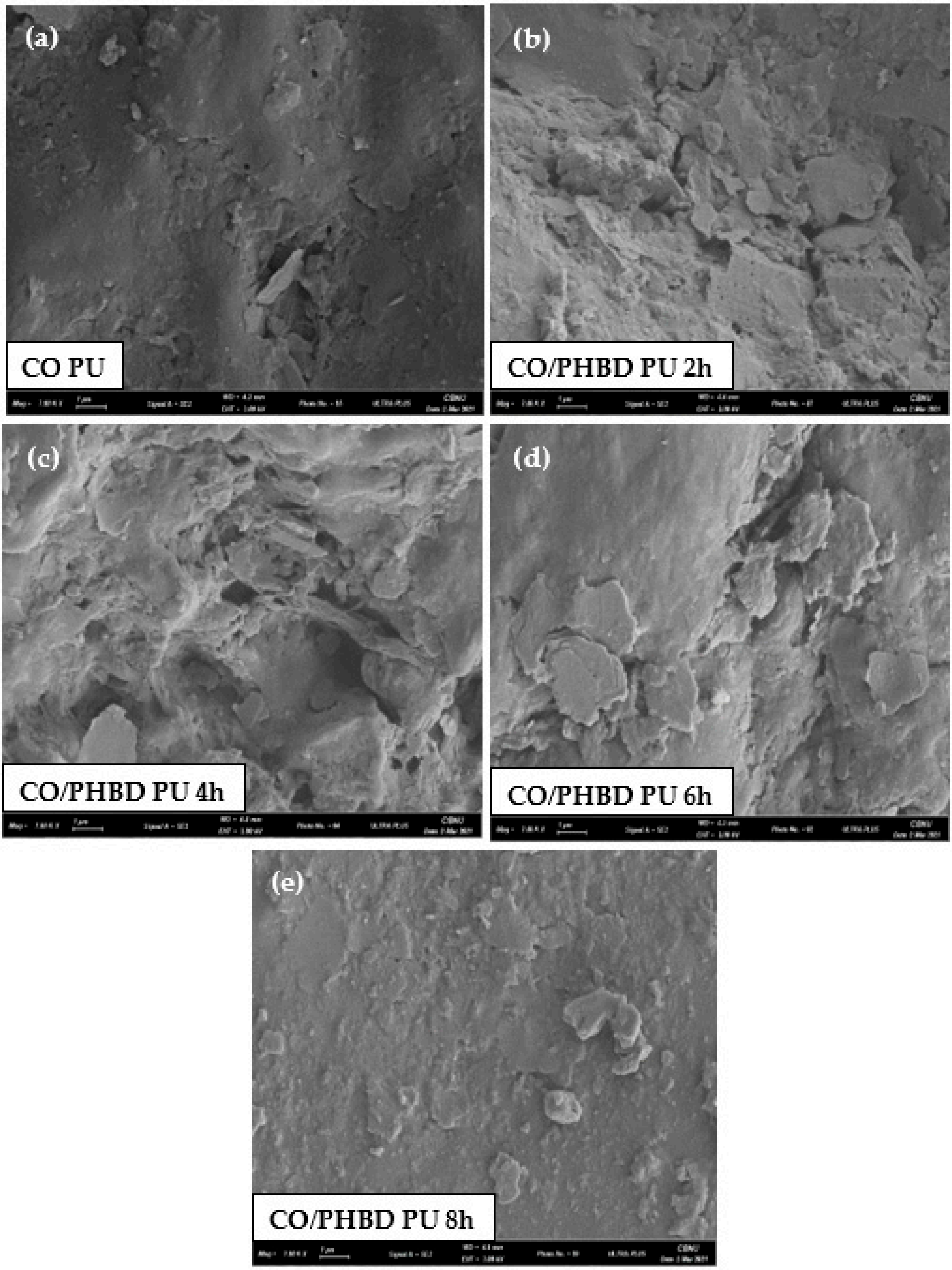

Figure S5. SEM analysis of CO/PHBD PUs after 35 days soil burial test.
